# Parallel detection of multiple biomarkers in a point-of-care-competent device for the prediction of exacerbations in chronic inflammatory lung disease

**DOI:** 10.1038/s41598-024-62784-8

**Published:** 2024-06-04

**Authors:** Niels Röckendorf, Katrin Ramaker, Karoline Gaede, Kristof Tappertzhofen, Lars Lunding, Michael Wegmann, Peter Horbert, Karina Weber, Andreas Frey

**Affiliations:** 1 Division of Mucosal Immunology and Diagnostics, Priority Area Chronic Lung Diseases, Research Center Borstel - Leibniz Lung Center, Member of Leibniz Health Technologies, Parkallee 1-40, Borstel, Germany; 2 BioMaterialBank-North, Department of Medicine, Research Center Borstel - Leibniz Lung Center, Parkallee 1-40, Borstel, Germany; 3 Division of Lung Immunology, Priority Area Chronic Lung Diseases, Research Center Borstel - Leibniz Lung Center, Member of Leibniz Health Technologies, Parkallee 1-40, Borstel, Germany; 4 Airway Research Center North (ARCN), German Center for Lung Research (DZL), Borstel, Germany; 5 Department of Spectroscopy and Imaging, Leibniz Institute of Photonic Technology, Member of Leibniz Health Technologies, Member of the Leibniz Centre for Photonics in Infection Research (LPI), Albert-Einstein-Straße 9, Jena, Germany

**Keywords:** Predictive markers, Cytokines, Asthma

## Abstract

Sudden aggravations of chronic inflammatory airway diseases are difficult-to-foresee life-threatening episodes for which advanced prognosis-systems are highly desirable. Here we present an experimental chip-based fluidic system designed for the rapid and sensitive measurement of biomarkers prognostic for potentially imminent asthma or COPD exacerbations. As model biomarkers we chose three cytokines (interleukin-6, interleukin-8, tumor necrosis factor alpha), the bacterial infection marker C-reactive protein and the bacterial pathogen *Streptococcus pneumoniae*—all relevant factors in exacerbation episodes. Assay protocols established in laboratory environments were adapted to 3D-printed fluidic devices with emphasis on short processing times, low reagent consumption and a low limit of detection in order to enable the fluidic system to be used in point-of-care settings. The final device demonstrator was validated with patient sample material for its capability to detect endogenous as well as exogenous biomarkers in parallel.

## Introduction

COPD and asthma are highly prevalent chronic respiratory diseases that affect hundreds of millions of people worldwide, not only in industrialized, but also in developing countries^1–3^. Both diseases are associated with a high degree of mortality and morbidity and, thus, cause major burden to public health systems. COPD and asthma are characterized by heterogeneous chronic airway inflammation and airway obstruction as well as by persisting respiratory symptoms such as shortness of breath^4^. Under treatment with long-acting β2-agonists and inhaled corticosteroids, or combinations of β2-agonists and muscarinic receptor antagonists, both diseases are usually well controlled^5^. However, acute aggravations of pulmonary inflammation and thus of symptoms, defined as exacerbations, still compromise patients’ quality of life, often require emergency hospitalization or intensive care treatment and in many cases even lead to death^6^. Early intervention in exacerbations can improve prognosis substantially. Consequently, a simple diagnostic system capable of predicting these events is highly desirable^7^. The earliest indicator for imminent exacerbations is the presence of an exogenous trigger that is capable to provoke an exacerbation. In asthma as well as COPD, most exacerbations are triggered by respiratory infections with e.g. rhinovirus (RV), respiratory syncytial virus or human metapneumovirus, or by bacteria including *Haemophilus influenzae*, *Streptococcus pneumoniae*, *Staphylococcus aureus* or *Moraxella catarrhalis*^8–10^. However, since not every respiratory infection results in an acute disease exacerbation, host factors are supposed to play a critical role in the pathogenesis of these events. Consequently, for reliable prognosis of exacerbations, endogenous biomarkers which are related to inflammatory conditions—such as interleukin- (IL-) 6, IL-8 and tumor necrosis factor alpha (TNF-α)—should be included in a predictive test system^11–15^.

Continuous and reliable monitoring of both, endogenous biomarkers and exogenous triggers, preferably in point of care (POC) or even home test diagnostic devices for daily use, can improve diagnostic accuracy: it will establish an individual baseline under stable conditions from which deviations could reveal developing exacerbations and indicate the necessity for therapeutic intervention at an early point in time. Such regular monitoring requires an easy-to-sample analyte that reflects both, the load of exogenous triggers and the level of endogenous biomarkers. We therefore aimed to develop a prospectively POC- and hometest-competent fluidic device that is capable of analyzing saliva from asthma or COPD patients for exacerbation-relevant markers and triggers. We ruled out a dip stick test format as this would have difficulties to deal with particulate analytes such as bacteria^16^, and PCR based methods as they are not suitable to analyze endogenous biomarkers in saliva. Instead, we decided to utilize antibody-based assays for our test system as those should be able to detect all types of analytes in a saliva sample, and developed robust assay protocols that are capable of being conducted in a fluidic cartridge system, with emphasis on quick results in POC or hometest environments.

The longterm goal of developing a POC or home test diagnostic device demands the transferability of the fluidic device into cost-effective mass production employing plastic materials and consisting of the least possible number of functional units. These components must be kept simple and standardized and should be easily implementable in existing fluid management systems. The fluid management in general should be robust, functionally reliable and safe to operate in POC or hometest settings. Only when a cost-effective, rapid and easy to handle solution is established, patient compliance will be high, allowing continuous monitoring to establish a personal baseline.

## Methods

### Materials

The following recombinant human proteins were used as model analytes: IL-6 (R&D Systems, Minneapolis, MN), IL-8 (Merck, Darmstadt, Germany), TNF-α (Sino Biological Inc., Wayne, PA) and C-reactive protein (CRP) (Sino Biological). *Streptococcus pneumoniae* strain R6 was employed as model pathogen (# BAA-255, ATCC, Manassas, VA). The following antibodies served as capture reagents: anti-IL-6 (clone MQ2-13A5; Biolegend, San Diego, CA), anti-IL-8 (clone H8A5; Biolegend), anti-TNF-α (clone MAb1; Biolegend) and anti-CRP (clone 108-2A2; antibodies online, Aachen, Germany). As detection reagents, the following biotinylated antibodies were applied: anti-IL-6 (clone MQ2-39C3; Biolegend), anti-IL-8 (clone E8N1, Biolegend), anti-TNF-α (Mab11, Biolegend) and anti-CRP (clone C7, antibodies online). For capturing of *S. pneumoniae* on slide surfaces, anti-phosphorylcholine IgA κ, (clone TEPC 15; Merck, Darmstadt, Germany) and for detection, biotinylated rabbit anti-*S. pneumoniae* antibody (MBS324036, MyBioSource, San Diego, CA) were used. Anti-FLAG M2 (Agilent, St. Clara, CA) served as setup/coupling control along with biotinylated FLAG peptide synthesized in-house following standard Fmoc procedures ^17^.

### Saliva sampling

Prospective anonymized collection of saliva samples from voluntary donors was carried out by the BioMaterialBank Nord at the Research Center Borstel, Borstel, Germany. All donors provided informed consent for participation. The study was approved by the ethics committee of the University of Luebeck (approval no. 16-167). All methods involving human material were carried out in accordance with relevant guidelines and regulations. Probands were divided into four groups according to their lung health status: asthma patients, COPD patients, subjects with an acute respiratory infection, and healthy controls. For the sampling procedure, all subjects were requested to refrain from food for 30 min and were asked afterwards to chew on paraffin chewing gum (GC Corp., Leuven, Belgium) for a minimum of 1 min. Saliva (total of 5 ml) was transferred to a polypropylene sampling vial. Samples were aliquoted, snap frozen in liquid nitrogen and stored at − 80 °C. Saliva was used for analysis either as individual samples, or as saliva pool obtained by mixing 1 ml saliva each from 30 randomly selected healthy controls. Cytokine levels in a subset of individual samples were measured using an enhanced cytometric bead array as a reference, following the manufacturer's guidelines (eCBA, Flex Set Kits; BD Biosciences, Franklin Lakes, NJ).

### Analyte detection on slide arrays in open well format using incubation chambers

For detection of endogenous analytes, arrays (2 rows of 8 arrays, each array 4 × 3 spots) of capture reagents were generated by depositing 20-nl droplets of the respective antibody solution (50 µg/ml (1 ng/spot) of anti-IL-8, anti-IL-6 or TNF-α in borate buffer (12.5 mM Na_2_B_4_O_7_, pH 8.5) onto 3D-Epoxy glass slides (PolyAn GmbH Berlin, Germany) using a nanoplotter (GeSiM GmbH, Dresden, Germany). As setup/coupling control, 50 µg/ml anti-FLAG was spotted in an analogous way. Slides were kept over night (o.n.) in a humidified atmosphere at 4 °C. On the next day, Grace Bio-Labs FlexWell™ grids (Merck) were attached to the slides thereby creating 2 × 8 well incubation chambers. The cavities were washed 3 × with 100 µl of PBST (0.1% Tween 20 (v/v) in 2.7 mM KCl, 1.5 mM KH_2_PO_4_, 136 mM NaCl, 8.1 mM Na_2_HPO_4_, pH 7.4). Slides were incubated for 2 h at room temperature (RT) with pooled saliva samples that had been spiked with analytes IL-8, IL-6 and TNF-α in a concentration of 10, 2 or 1 ng/ml each, washed 3 × with 100 µl of PBST and incubated with 50 µl of biotinylated detection antibodies for 90 min at RT. Slides were washed 5 × with 100 µl of PBST and incubated with 50 µl of 1 µg/ml streptavidin Alexa 680 (Thermo Fisher Scientific, Waltham, MA, USA) in PBST for 1 h at RT. After washing 5 × with PBST, the slides were dried and the fluorescent signal was recorded at 700 nm using a microarray imager (Odyssey CLx, Li-Cor Biosciences, Lincoln, NE, USA).

For detection of *S. pneumoniae* on slide surfaces, arrays were generated on 3D-Epoxy glass slides by depositing 20-nl droplets of *S. pneumoniae* capture antibody in patterns of 4 × 3 spots (5—20 ng/spot). Slides were kept o.n. at 4 °C and equipped with Grace Bio-Labs incubation chambers as above, wetted for 30 min with 50 µl/cavity of PBST and washed 3 × with PBST (75 µl/cavity). Slides were incubated for 1 h at RT with 50 µl of PBST or pooled saliva samples spiked with *S. pneumoniae* at varying concentrations (1 × 10^5^ CFU/ml to 1 × 10^8^ CFU/ml), or saliva devoid of bacteria. Slides were washed 3 × with PBST, incubated with 50 µl of biotinylated detection antibody for 90 min at RT, washed 5 × with PBST and incubated with 50 µl of 1 µg/ml streptavidin-Alexa 680 in PBST for 1 h at RT. After washing 5 × with PBST the slides were dried and the fluorescent signal was measured as above.

### Analyte detection on slide arrays using fluidic devices

The main fluidic components of the detection devices as well as the incubation chambers were manufactured in an Agilista 3200W 3D printer (Keyence Deutschland GmbH, Neu-Isenburg, Germany) using AR-M2 printing material. Dimensions of each analysis chamber were 14 mm × 3 mm × 0.2 mm, generating a chamber volume of 8.4 µl; the total volume of the complete fluidic system was ~ 20 µl. For the parallel detection of analytes in version 1 of the fluidic device (quadriplex design), four arrays of capture reagents were generated on 3D-Epoxy glass slides by depositing 20-nl droplets of the respective capture antibody solutions in duplicate spots, with 1.5 mm spot-to-spot distance. Depending on the experimental set-up, a varying combination of four different capture antibodies was used (anti-IL-6, anti-IL-8, anti-TNF-α, anti-CRP each at 1.5 ng/spot, anti-phosphorylcholine IgA κ at 5 ng/spot). Slides were incubated o.n. in a humidified atmosphere at 4 °C, dried and stored at 4 °C under argon atmosphere for a maximum of 72 h. For application, slides were mounted onto the 3D printed incubation chambers by using a dual adhesive foil. All subsequent washing steps were carried out by slowly pushing the respective washing buffer via a luer-fitted 1-ml syringe over each individual analysis chamber. Likewise, samples and detection reagents were applied through the luer ports with the same syringe. Chambers were washed 3 × by flushing with 200 µl of PBST, filled bubble free with 150 µl of saliva sample and incubated for 1 h at RT. After a second washing step (3 × 200 µl of PBST), the respective biotinylated detection antibodies were applied (one individual detection antibody for each array) and incubated for 1 h at RT. Chambers were washed again (5 × 200 µl of PBST) and subsequently incubated with 150 µl of 1 µg/ml streptavidin-Alexa 680 in PBST for 1 h at RT. Chambers were flushed again (5 × 200 µl of PBST), liquids were removed and the chambers were imaged at 680 nm excitation and 700 nm emission wavelength in a microarray imager (Odyssey CLx, Li-Cor Biosciences, Lincoln, NE) at 21 µm resolution, using the built-in solid state diode laser for excitation and silicon avalanche photodiodes for detection. Under these conditions, autofluorescence and background signals from the microfluidic device were negligible.

For detection of IL-8 in version 2 of the fluidic device (parallel design), four capture reagent arrays of 8 spots each were generated on slides using anti-IL-8 capture antibody at 1.5 ng/spot. Slides were mounted onto the 3D printed channel incubation chamber (200 µm depth), and all chambers were washed as described above. The chambers were then filled bubble free with 400 µl of pooled saliva samples spiked with 10 ng/ml IL-8 and incubated for 1 h at 34 °C. After a second washing step (3 × 500 µl of PBST) biotinylated anti-IL-8 detection antibody was applied to all chambers and incubated for 1 h at 34 °C. Chambers were washed 5 × by passing 500 µl of PBST through the chambers and incubated with 400 µl of 1 µg/ml streptavidin-Alexa 680 in PBST for 1 h at RT. Chambers were again washed 5 × with 500 µl of PBST, liquids were removed and fluorescence was measured as above.

## Results and discussion

The desired user-friendly test-system requires an assay format that allows high sensitivity and specificity along with fully automated sample handling, analyte detection and data interpretation. Consequently, our focus was laid on the design of the fluidic system and the translation of classical bioassay protocols to a unit into which all relevant process steps of the analytical protocol can be implemented. This includes facile sample supply with biofluids that contain the relevant biomarkers, fluid exchanges to remove sticky biological compounds, and the fluorescence-based detection of bound analytes. Handling of sample materials, reagents or washing fluids was kept as simple as possible in all processing steps.

### Hardware design

**Figure 1:**
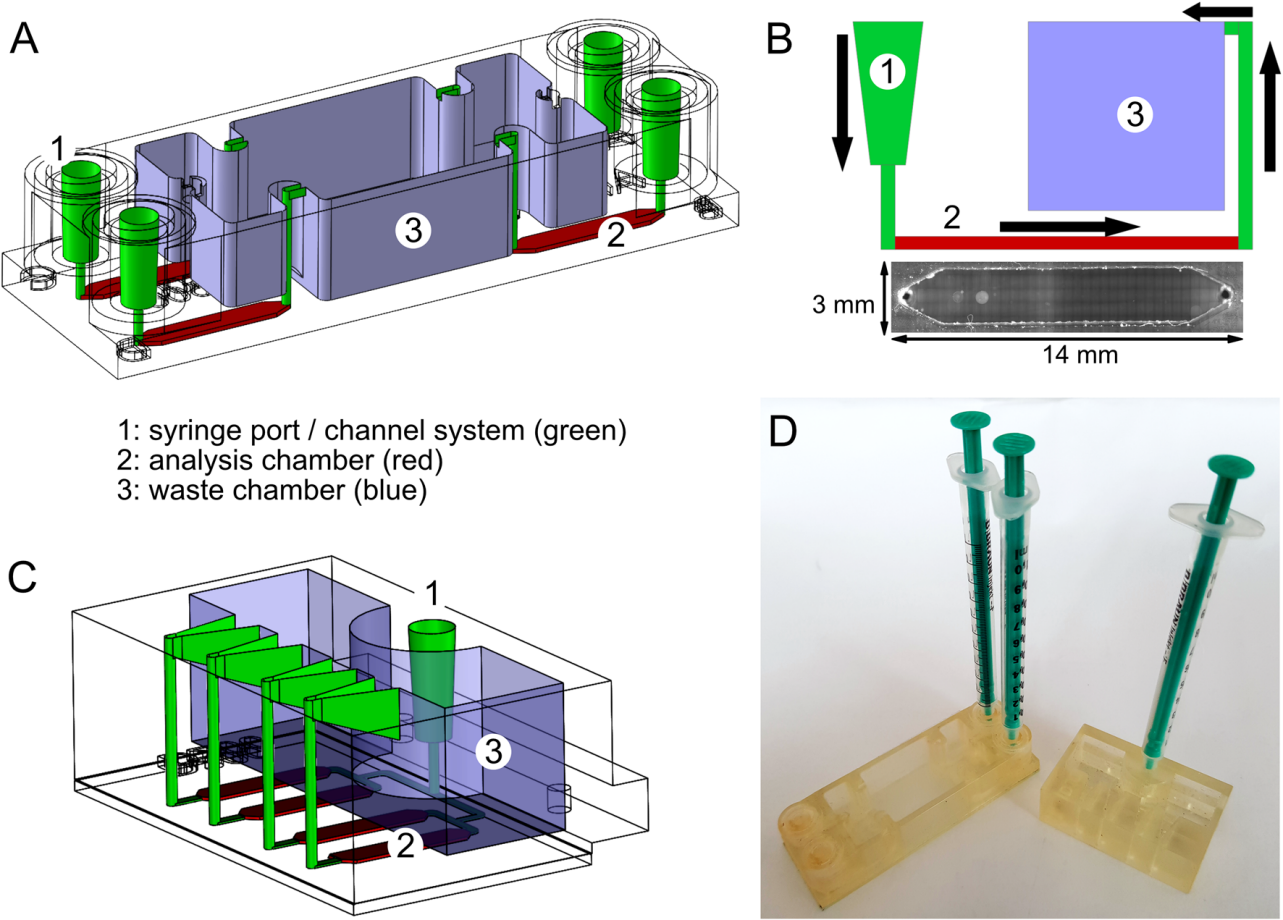
Design of the microfluidic reaction device. (**A**) Device version 1: four chambers loaded/processed individually; (**B**) Scheme of fluid flow and microscopic top view of individual analysis chamber (grayscale image); (**C**) Device version 2: four chambers loaded/processed in parallel; (**D**) Demonstrator units of fluidic devices version 1 and 2, equipped with syringes for manual operation.

The initial version of the fluidic device was a quadriplex system, comprising four separate analytical chambers each equipped with individual inlet port and outlet channel connected via separate fluid conduits (Fig. 1A). The 3-D manufactured top piece was designed with a footprint of 25 mm × 75.5 mm and a height of 14 mm. This top piece can be mounted onto standard glass slides which will then close the fluid coves and create the analysis chambers as well as a waste reservoir with a capacity of up to 9 ml volume. Syringe (luer) ports were integrated as loading connectors to allow manual operation. A pivotal issue in choosing the dimensions for the analysis chambers was the consideration of shear stress caused by the fluid streaming over solid surfaces where analyte-capturing ligands are immobilized. On the one hand, smaller fluid layers will cause larger shear forces since fluids move faster with decreasing conduit diameter, in particular when the parabolic flow profile of laminar fluid movement switches into the turbulent state. In such a case, larger analytes such as viruses or bacteria might detach from the surface due to fluid friction as shear forces are large compared to the binding forces of the small contact spots between analyte and surface-bound capture reagent. On the other hand, a slow fluid movement in the chamber facilitates air bubble formation which will interfere with the readout. Moreover, a switch from turbulent to laminar flow increases diffusion times of the analytes to their respective ligands. In light of those interdependent variables we sought an empirical solution to the problem. Top pieces that allowed chamber heights from 0.2 to 2 mm were 3-D printed and investigated in different analytical settings. We found that a chamber height of 200 µm performed best with the analyte solutions tested. Consequently we chose the following dimensions for the analysis chamber: length, 14 mm, width, 3 mm and height, 0.2 mm, resulting in a total volume of 8.4 µl (Fig. 1B).

To ensure robust operation, tube diameters were set to 1 mm. As can be seen in Fig. 1B, the interface between the adhesion foil and the glass surface is pervaded by small gas-filled cavities, but these were found to be not critical to the biochemical assays performed in the chambers. Apparently, flow rates were low enough to prevent high pressure in the chambers, and no fluid leaked into the offline cavities, such that no background fluorescence caused by entrapped detection reagents was observed. Air bubbles could be easily driven out from all parts of the chamber and detrimental surface effects such as trapping of air did not occur. As autofluorescence of the AR-M2 material was low in the far-red spectral range at 700 nm, Alexa-680 labeled streptavidin was readily detectable without perceptible background noise.

As an alternative to the quadriplex design, the fluidic device was laid out for parallel operation (Fig. 1C). This device has a reduced footprint of 46 × 32 mm and a height of 16 mm. While the geometry for each analysis chamber remained unchanged, arrangement of the chambers was modified together with the channel structures. The aim of these modifications was to achieve uniform filling and emptying of all chambers. For the planned integration into an automated cartridge system, fluidic uniformity is an essential requirement. Figure 1D shows demonstrators of both versions of the analytic device, fitted for manual operation. Based on these designs, secondary functional elements, such as fluid reservoirs and a suitable fluid management system, can be integrated in a later step.

### Analyte and biomarker selection

For complex disorders such as chronic airway inflammation a battery of biomarkers must be monitored in order to gain the desired diagnostic power. In addition, the use as POC or hometest settings requires noninvasive, rapid acquisition of sample material that is available in sufficient amounts. Saliva meets these requirements, and it has been shown that a wide variety of endogenous and exogenous biomarkers can be present in this sample material^18^. Importantly for diagnostic measures in inflammatory lung diseases, the analyte is released into the oral cavity which is anatomically linked to the site of inflammation and thus can pick up biomarkers liberated onto the mucosa at inflamed sites. Indicative for inflamed tissues are proinflammatory cytokines such as IL-6, IL-8 and TNF-α^11–14^. In case of a microbial infection, CRP is a known endogenous biomarker^19,20^. As an analyte representing the exogenous biomarkers, we selected the bacterium *Streptococcus pneumoniae* as prototypic airway pathogen.

### Assay design

For the selected biomarkers, detection reagents are commercially available and laboratory methods for their detection have been described. We improved typical protocols for e.g. microtiter plate-based biological assays to shorten incubation times and to allow rapid and sensitive readout. As assay principle we chose the sandwich immunoassay format with analyses to be carried out in parallel for the different markers (for details, see Fig. S1 in Supporting Information).

The assays were performed on slide arrays for which glass slides functionalized with epoxy groups on their surface were commercially available. These slides are also equipped with a thin layer of hydrophilic polymers which minimizes unwanted attachment of (hydrophobic) biomolecules onto the surface. Functionally proven compounds were employed as capture, detection and signal amplification reagents. In all set-up variations, arrays of the respective capture antibodies were first spotted onto the slide forming covalent links with the functionalized surface. Captured analytes were detected with specific, biotinylated antibodies, followed by fluorophore-labeled streptavidin, and fluorescence signals on the processed slides were recorded in a microarray imager.

### Detection of disease markers in an open well micro-device

**Figure 2:**
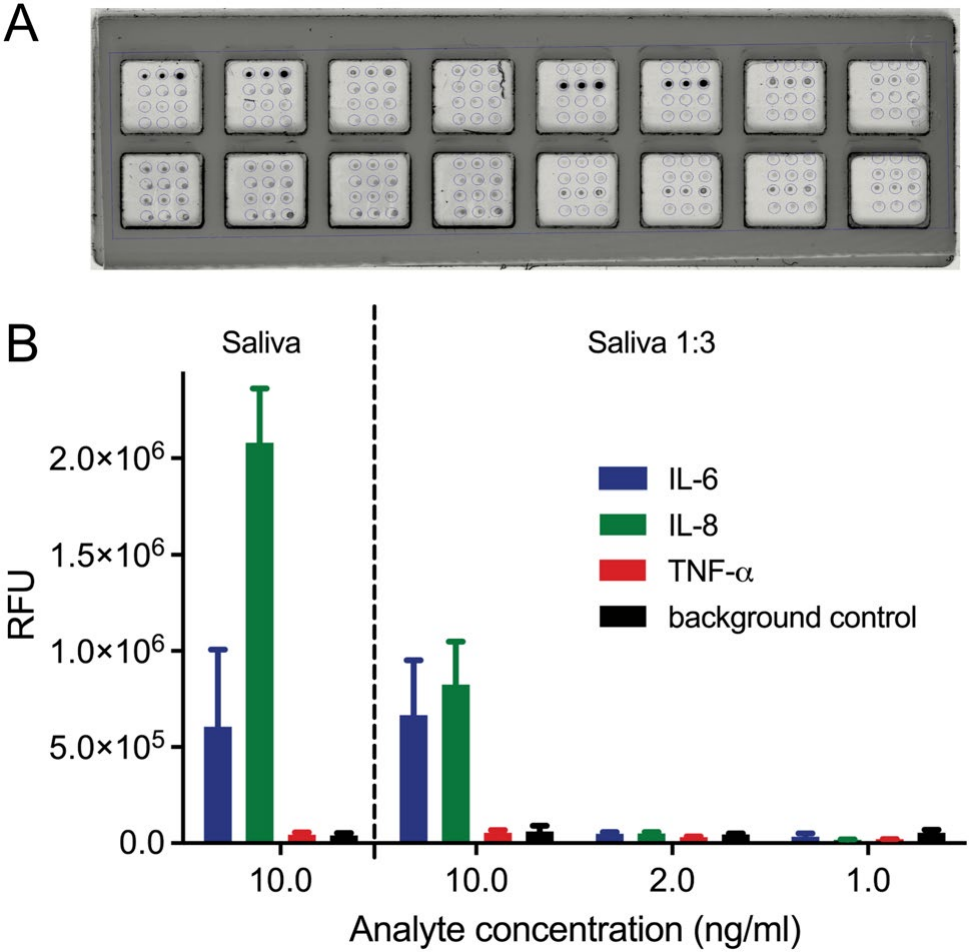
Detection of endogenous biomarkers by sandwich immunoassay. (**A**) Slide arrays processed in open well format. (**B**) Measurement of IL-6, IL-8 and TNF-α at different concentrations in undiluted or diluted saliva (N = 3).

We first designed an open well slide system for performing the immunoassays. Array dimensions on the slides were chosen to accommodate commercially available grids which were fixed on the slide surface thereby providing specified reaction cavities that were loaded and processed manually. This set-up was used to optimize reagent concentrations and ratios, and to investigate the effect of saliva as biological matrix on the traceability of the different analytes. For detection of endogenous analytes, saliva samples were spiked with the different cytokines at varying concentrations, with undiluted saliva and saliva diluted 1:3 in PBS buffer being used as sample matrix. While IL-6 and IL-8 were easily detectable at an analyte concentration of 10 ng/ml, for TNF-α only a very weak signal was obtainable (Fig. 2).

The presence of saliva matrix had no effect on the detectability of IL-6 as signal intensities were identical in undiluted and in diluted saliva. For IL-8, the signal was even higher in the spiked undiluted saliva than in diluted matrix. This may be due to the presence of endogenous IL-8 in the used saliva pool which may contribute to the higher signal in the undiluted sample (see below). Despite those differences between the individual cytokines, detectability of the respective analytes was considered sufficient to be verified in the fluidic chamber system after adaptation of the procedure.

**Figure 3:**
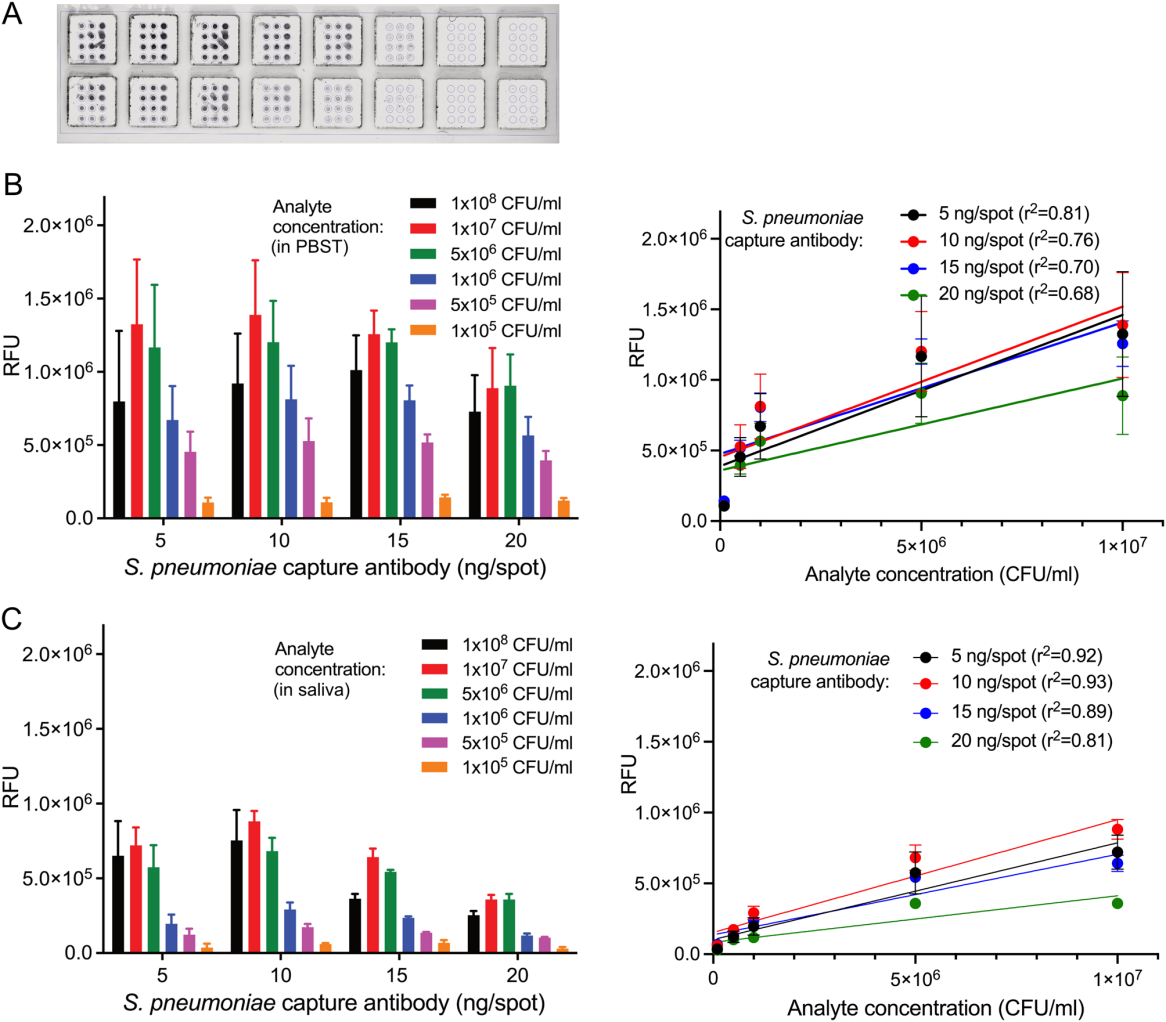
Detection of model pathogen *S. pneumoniae.* (**A**) Slide arrays processed in open well format. (**B**, **C**) Measurement of increasing numbers of bacteria, captured by different amounts of antibody in individual spots. Analyte was applied either in PBST (B) or in saliva (**C**) (each N = 3). Graphs in the right panel depict results of linear regression analysis assessing the relationship between fluorescence read-out (relative fluorescence unists, RFU) and analyte concentration in the samples. Note that the highest concentration of bacteria (1 × 10^8^ CFU/ml) has been omitted from all regression analyses.

To include exogenous triggers of exacerbations in our system we selected a capture antibody that should be able of reacting with a broad variety of microorganisms: an antibody directed against phosphorylcholine, which is an essential component of cellular membranes in most bacterial species^21–23^. The specificity of the system would be provided by the detection antibody, in our case an antibody which recognizes numerous *S. pneumoniae* serotypes. In order to evaluate the assay setup for the detectability of *S. pneumoniae* on glass surfaces, arrays were generated with different amounts of capture antibody and probed with bacteria at different concentrations (Fig. 3).

The bacteria were well detectable in our set-up, but detection quality varied depending on the test conditions. Regression analysis showed good correlations between analyte concentration and signal intensity, as long as the highest bacterial concentrations were omitted from the calculations (Fig. 3, right panel). Hence, up to a concentration of 1 × 10^7^ CFU/ml, the increasing amounts of bacteria were well reflected by the signal intensities obtained in our detection system, but higher concentrations tended to be underestimated. Likewise, the detectability of the bacteria varied in dependance of the amount of capture antibody per spot, although the differences were generally not statistically significant (One-way ANOVA followed by Friedman multiple comparison test). Over all, the highest signals were obtained with 1 × 10^7^ CFU/ml of bacteria at 10 ng of capture antibody. Neither higher amounts of capture antibody nor of bacteria led to an increase in signal intensity. We attribute this “detectability optimum” to the fact that *S. pneumoniae* is a huge antigen and binding to it is limited by sterical constraints.

In contrast to the detection of cytokines, the sample matrix did influence the detectability of *S. pneumoniae*, as signals were reduced when the analyte was applied in saliva in comparison to buffer as matrix. These differences were statistically significant for nearly all bacterial concentrations measured in combination with capture antibody amounts of 15 ng/spot and 20 ng/spot, and for the lower bacterial amounts captured with 5 ng antibody/spot (*p* < 0.05, unpaired t-test with Welsh's correction). However, they did not differ significantly between saliva and PBST with any of the bacterial concentrations when 10 ng capture antibody per spot were used, which confirms this coating concentration as optimal for the assay set up.

### Detection of disease markers in a quadriplex chamber microfluidic device

**Figure 4:**
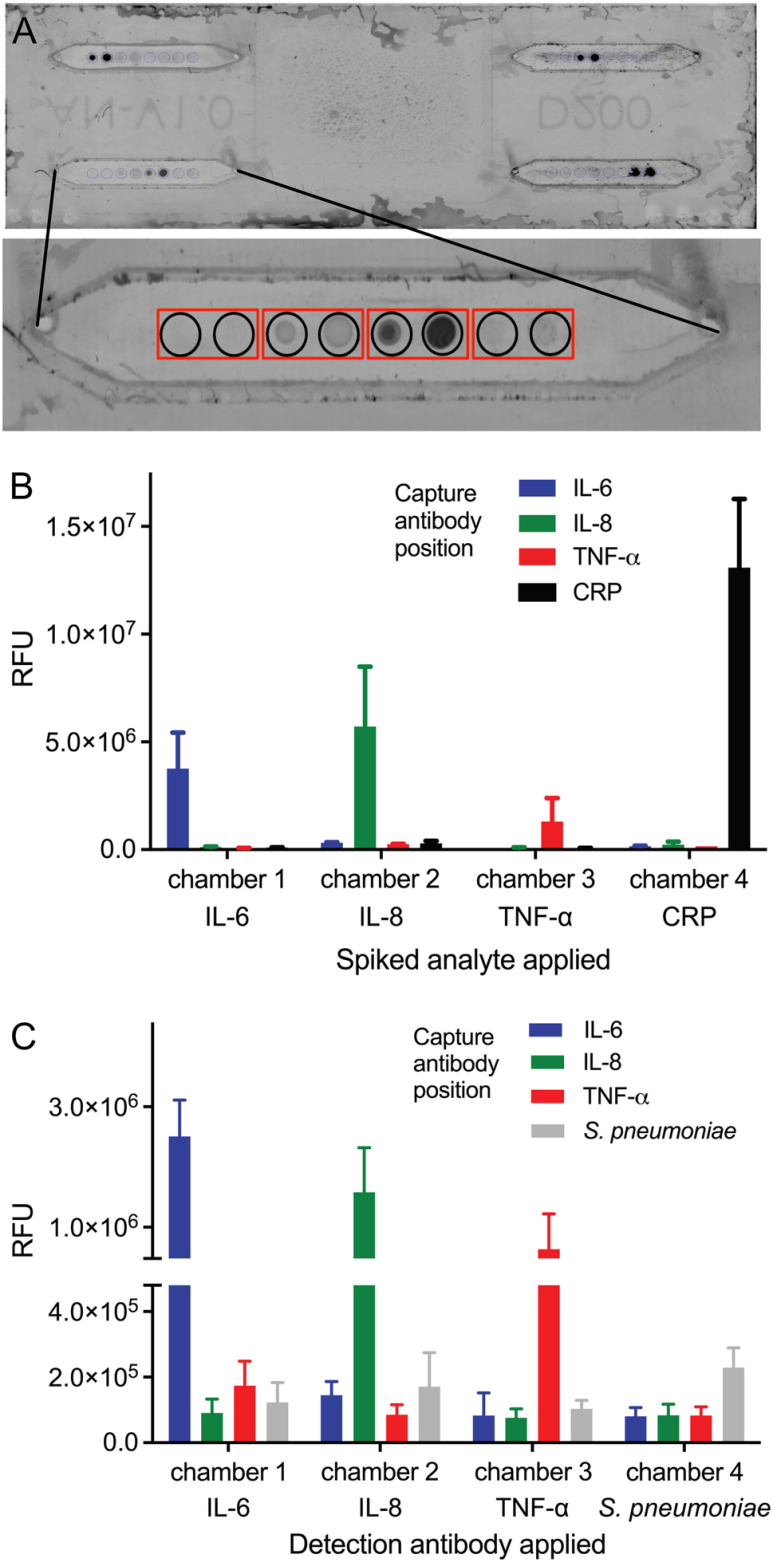
Detection of markers on glass slides using a microfluidic device. (**A**) Slide with four reaction arrays carrying duplicate spots of capture antibodies for the detection of four different analytes. (**B**) Detection of analytes in individually spiked saliva samples (analyte concentration: 10 ng/ml) using a mixture of biotinylated detection antibodies. (**C**) Detection of analytes in mixed spiked saliva samples (IL-6 & IL-8: 10 ng/ml, TNF-α: 25 ng/ml, *S. pneumoniae*: 2 × 10^5^ CFU/ml) using individual biotinylated detection antibodies in each reaction chamber.

The next steps aimed to transfer the assays from open-well format to the microfluidic device. The first experiments were conducted with the quadriplex chamber system (Fig. 1A). For parallel detection of four analytes in this device, two different reaction set-ups were tested. In each case, four arrays of capture antibodies (duplicate spots for each analyte to be tested, resulting in eight spots per reaction area) were immobilized on the glass surface in the predefined reaction areas (Fig. 4A). After mounting the slides into the reaction device, two different set-ups were chosen to gain maximum information on potential cross-reactivities and interferences between the different analytes and detection reagents: either each reaction chamber received a saliva sample spiked with one individual analyte only, and all four chambers were subsequently treated identically for analyte detection with a mixture of all four anti-analyte antibodies (Fig. 4B), or, all four reaction chambers were incubated with saliva samples spiked with a mixture of the four different analytes, and analyte detection was achieved with one specific anti-analyte antibody in each of the four reaction chambers (Fig. 4C).

All endogenous analytes, which had been added to the saliva matrix in a concentration of 10 ng/ml respectively 25 ng/ml (TNF-α in set-up 2), were readily detectable in either setup, with signals well above background and signal-to-noise ratios around 5. Thus, even lower spiking concentrations such as 1 ng/ml should be feasible. This would translate into limits of detection (LODs) of about 40–100 pM analyte, which is in accordance with previous reports where LODs in the picomolar to low nanomolar range were observed for fluoroimmunoassays (FIA) conducted on plain surfaces^24,25^.

For *S. pneumoniae*, the signal was rather poor in saliva when compared to phosphate-buffered saline, perhaps due to masking of capture and detection antibody binding sites by saliva-derived anti-*S. pneumoniae* immunoglobulins. Cross reactivities of the detection antibodies with the different analytes were minimal, and despite the high spiking concentrations no false positive signals for other analytes were observed.

### Quality control I—fluidic and detection reagent robustness

**Figure 5:**
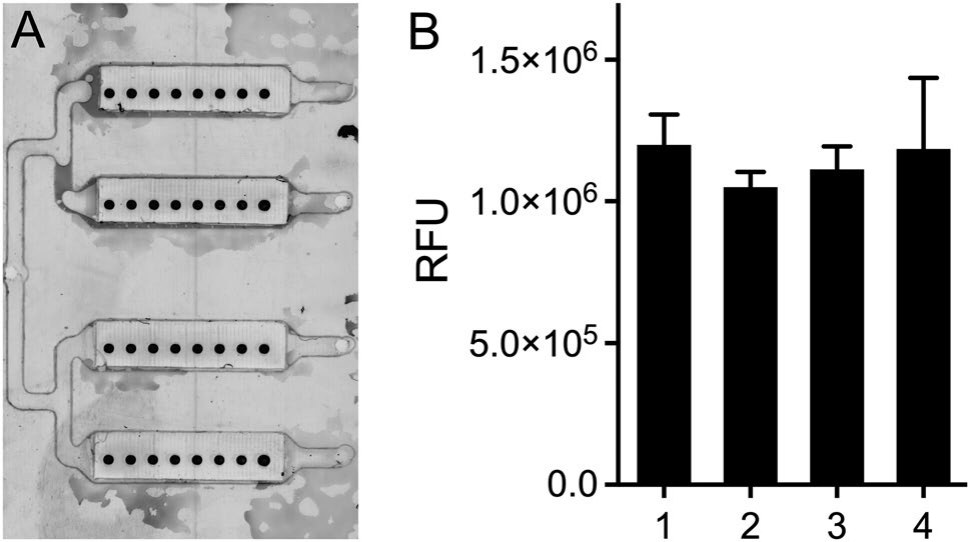
Measurement of IL-8 in a fluidic device with 4 parallel channels attached to a single sample inlet structure (**A**). The measurements of IL-8 are not significantly different (1way ANOVA, Kruskal Wallis Test, Dunn’s multiple comparisons test) in the four channels (**B**).

The sturdiness of assays in a branched channel system was investigated using the advanced 3D printed fluidic device (Fig. 1C) with four chamber structures connected to a single sample inlet port (Fig. 5A).

Measurement results in the four chambers were comparable, differences between the mean signal intensities obtained in the different chambers were not significant (Fig. 5B, ANOVA, Kruskal Wallis Test, Dunn’s multiple comparison test) indicating that our design ensures simultaneous and uniform filling of the chambers from one fluid inlet port onwards. This is of special concern with regard to a later automatization of the fluidic system and an important requirement for further development of the device towards POC- or home use.

### Quality control II—detection limits for biomarkers in patient samples

To validate the biomarker detection system under real-life conditions, we analyzed individual human saliva samples for the content of the endogenous biomarkers in our microfluidic device. Samples were obtained from a study cohort that enrolled patients with either asthma or COPD, healthy individuals and people suffering from an acute respiratory infection. None of the patients with asthma or COPD exhibited an acute disease exacerbation at the time of sampling. Nine saliva samples were arbitrarily selected from each group and not treated further. To assess matrix effects, a tenth sample of each group was spiked with the recombinant cytokines used before, at a nominal concentration of 1 ng/ml each. In order to obtain reference values for the cytokines IL-6, IL-8 and TNF-α in human saliva, all samples were analyzed using an enhanced cytometric bead array (eCBA) (see Supporting Information for listing of individual values for all samples).

In the spiked saliva samples, the concentration of IL-8 determined by eCBA was in all cases higher than the added amount of 1 ng/ml, most likely due to the presence of endogenous IL-8 in the sample. In contrast, the measured concentrations of IL-6 and TNF-α were much lower, displaying values between 12 and 40% of the added amount. Diminished detectability in saliva has been reported for these two cytokines in previous studies, with recovery rates of 50–80% for IL-6 and of about 44% for TNF-α^26,27^. While this might indicate that the recombinant proteins used for spiking are not as traceable in the samples as their human counterparts, it can also signify that they are degraded or masked in saliva.

In the non-spiked saliva samples, the concentrations of endogenous IL-6 and TNF-α were very low (in nearly all samples < 50 pg/ml), well below the levels detectable by the fluoroimmunoassay setup in our experimental devices. IL-8 concentrations in non-spiked saliva samples were considerably higher, ranging from 0.1 ng/ml to nearly 20 ng/ml. The highest amounts of IL-8 were in general present in saliva from COPD patients (Table 1).

**Table 1:** Salivary cytokine levels in humans with inflammatory airway disorders versus healthy individuals.

Cytokine	Health status	Mean ± SD	Median	Min	Max	CV
		pg/ml				%
IL-6	Healthy	6.35 ± 5.91	4.26	0.39	18.8	93.1
	Infection	3.96 ± 2.94	3.42	0.90	9.09	74.2
	Asthma	6.78 ± 6.66	4.20	0.53	21.8	98.3
	COPD	12.5 ± 13.4	5.66	0.62	35.6	107
IL-8	Healthy	1248 ± 839	1073	353	2759	67.2
	Infection	786 ± 573	588	202	1716	72.9
	Asthma	1792 ± 2018	1312	283	6686	113
	COPD	5666 ± 7801	1676	104	19,214	138
TNF-α	Healthy	12.9 ± 10.5	11.1	0.00	34.6	81.0
	Infection	5.50 ± 4.17	4.68	1.23	12.3	75.9
	Asthma	12.6 ± 20.9	6.30	0.00	65.4	166
	COPD	26.8 ± 43.8	10.7	0.51	137	163

For each cytokine, minimum and maximum values as well as coefficient of variation (CV) in each group are given. No statistically significant differences were found between the health status groups (*p* > 0.05; ANOVA, Kruskal Wallis tests); SD: standard deviation; CV, coefficient of variation.

Notably, significant differences were not detectable between healthy and diseased groups for any of the investigated salivary cytokines; in fact, a very high variability in the cytokine levels existed between individuals within each group, which is illustrated by the high coefficient of variation (CV). This underscores the necessity to determine individual baselines for each patient, which translates into the need for routine use of such a point-of-care device by patients with asthma and COPD.

**Figure 6:**
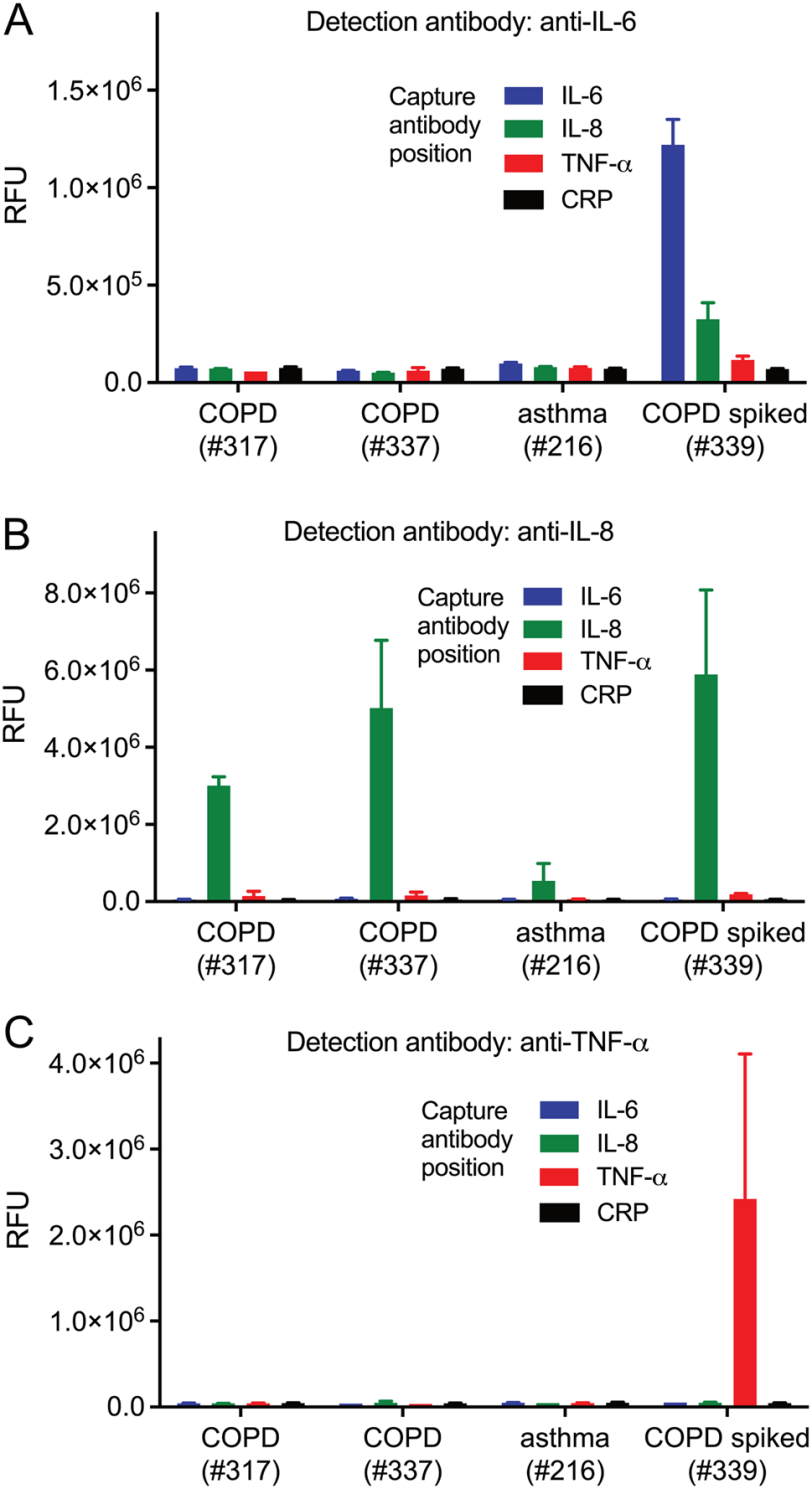
Detection of endogenous biomarkers IL-6, IL-8 and TNF-α in non-spiked saliva samples of selected asthma- and COPD patients, using the quadriplex fluidic device. One saliva sample spiked with IL-6, IL-8 and TNF-α, at 10 ng/ml each, was used as control.

In order to find out whether our current setup provides sufficient sensitivity, four patient samples containing high cytokine levels (one from the asthma group, two from the COPD group and one spiked sample) were analyzed in the quadriplex fluidic device. IL-6 and TNF-α were detectable in the spiked sample only, whereas IL-8 was detected in all samples (Fig. 6).

Over all, our device did not reach eCBA sensitivity. The better performance of the bead-based assay may be explained by the larger surface area of a spherical carrier compared to a flat spot, and to higher quantum yield and extinction coefficient of the eCBA-fluorophore (R-phycoerythrin) compared to Alexa 680 which we used. This might result in a lower sensitivity of our setting compared to the eCBA, and might explain why only IL-8, of which the concentration in saliva is more than tenfold higher than that of IL-6 and TNF-α, was detectable in non-spiked samples. It is therefore mandatory to improve the sensitivity in our system so that it can be used in future POC devices for the detection of IL-6 and TNF-α in saliva as well. While the incorporation of a signal amplification step, employing e.g. catalyzed reporter deposition^28^ or cation exchange reactions in ionic nanocrystals^29,30^ for fluorescence enhancement, or the use of high quantum yield fluorophores such as quantum dots^31^ is a worthwhile consideration, these changes would require substantial modifications in the assay set-up and in the detection reagents used. Ultimately, the choice of the readout system is likely the most crucial step. In the recent past, advances in both, sensitive optical and electronical detection systems have been reported. On the optical side, femtomolar concentrations (1 pg/ml) of IL-6 were detectable by combining drop-coating deposition Raman spectroscopy and graphene-enhanced Raman spectroscopy^32^, and even lower, zeptomolar concentrations of serum IL-6 were detected by evanescent field-enhanced fluorescence imaging^33^. Salivary IL-8 could be sensed fluorescently with a confocal optics based sensor at low femtomolar concentration^34^. Yet, although of impressive sensitivity, such optical sensorics still require extensive readout hardware which is not practical for implementation in a POC system.

Less hardware demanding are electronic sensor systems. Here, different readout systems based on e.g. amperometry, conductometry, voltametry, or photoelectrochemical approaches utilizing optical properties of semiconductors are possible. For cytokine detection, mostly electrical impedance spectrometry (EIS)^35–37^ and field-effect transistors (FET)^38–40^ have been used, mainly with serum samples. Lower detection limits ranged from low attomolar concentrations (10 ag/ml) for IL-6^35^ to low femtomolar concentrations for IL-8 and TNF-α (6 fg/ml and 0.1 pg/ml, respectively)^36,39^, thus, well below the cytokine concentrations found in our clinical samples.

As yet, none of the reported high-end read-out designs, neither optical nor electronical, combines such a sensitivity with multiplexing and the use of saliva as sample matrix. Once moderately sized and -priced detection hardware becomes available, it can be merged with the immunochemical layout and a miniaturized fluidic design as described here by us. Such sensoric systems shall allow creation of easy-to-use point-of care and point-of need analytical devices for the detection of upcoming exacerbations in patients with asthma and COPD.

## Conclusions

This work provides a proof-of-concept for multiplex saliva cytokine diagnostics that shall be suitable for the detection and prediction of upcoming exacerbations of stable asthma and COPD. It reveals the constraints and demands for next generation analytical systems suited for salivary cytokine monitoring. The clinical finding that individual variation in salivary cytokine levels is higher than expected, preventing group-based comparisons of different health conditions, asks for personal cytokine baselines and thus for translation of such devices into cheap and easy-to-use point-of-need test systems which a patient can use routinely at home.

### Supplementary Information

Supplementary Information.

## Data Availability

All original data generated/analyzed in this study are available from the corresponding author on reasonable request.
