# Supplementary material for: Parallel detection of multiple biomarkers in a point-of-care-competent device for the prediction of exacerbations in chronic inflammatory lung disease
Source: Sci Rep. 2024 Jun 4;14:12830. doi: 10.1038/s41598-024-62784-8 (PMC11150478; doi:10.1038/s41598-024-62784-8)
Supplement: Supplementary file 1 — Supplementary Information. [file 41598_2024_62784_MOESM1_ESM.pdf]

Supporting Information:
Parallel detection of multiple biomarkers in a point-of care-competent device for the prediction of exacerbations in chronic inflammatory lung disease.

Niels Röckendorf, Katrin Ramaker, Karoline Gaede, Kristof Tappertzhofen, Lars Lunding, Michael Wegmann, Peter Horbert, Karina Weber, Andreas Frey

Supporting Figure S1: Capture assay set-up for the detection of different analytes

A Coupling of capture antibody onto oxirane-modified glass surface

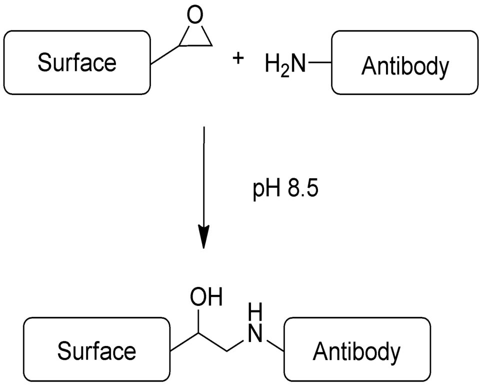

B Immobilization of analytes in different areas of glass slides via specific capture antibodies and detection by biotin-labeled antibodies and fluorophore-labeled streptavidin

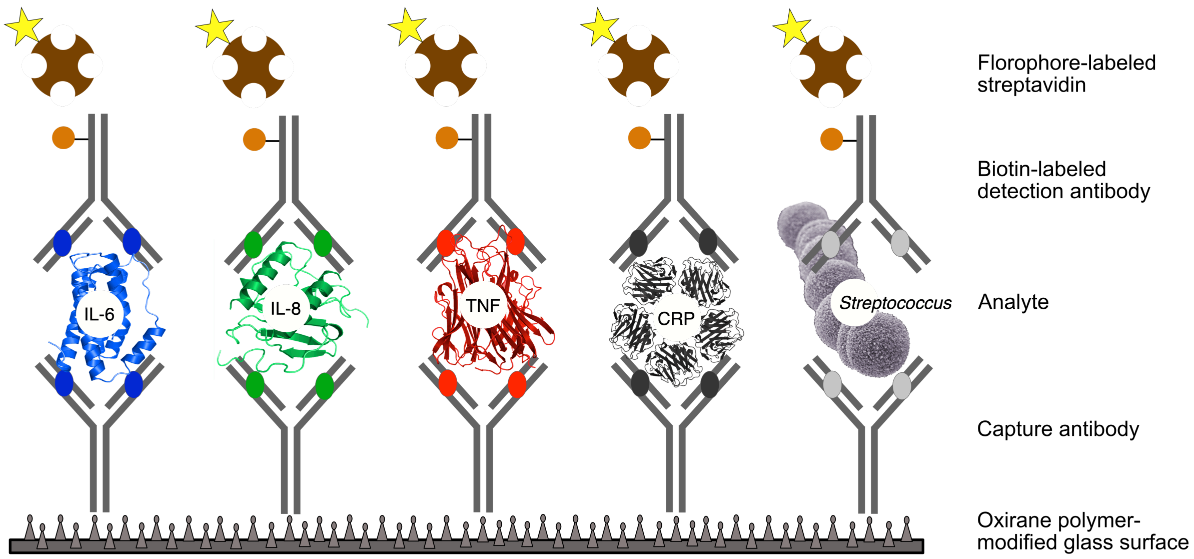

Supporting Table 1: Cytokine levels in saliva samples of asthma- and COPD patients as well as samples from healthy control subjects and control subjects with airway infections as measured by cytometric bead array (eCBA) test. Spiked Samples (each cytokine added in a nominal concentration of 1000 pg/ml) are marked by *. Saliva samples analyzed in the fluidic device demonstrator (Figure 8 in main manuscript) are indicated by bold-faced numbers.

**Sample ID**	**Group**	**IL-6 [pg/ml]**	**IL-8 [pg/ml]**	**TNF-α [pg/ml]**
39*	healthy	116.89	1666.65	267.89
49	healthy	18.77	1339.19	34.59
59	healthy	0.39	510.81	5.49
61	healthy	4.67	353.01	3.67
62	healthy	4.26	1625.87	16.24
68	healthy	3.93	1072.67	21.79
77	healthy	3.23	926.53	10.75
78	healthy	7.80	2759.22	11.12
79	healthy	1.40	399.00	0.00
80	healthy	12.72	2248.26	12.49
124	airway infect	9.09	1636.91	12.34
138	airway infect	3.42	1043.61	4.68
139	airway infect	4.43	283.09	2.05
158	airway infect	0.90	588.08	7.54
160*	airway infect	153.90	1786.36	419.10
161	airway infect	8.46	850.62	7.00
162	airway infect	1.79	377.82	1.23
166	airway infect	3.62	1715.70	10.90
172	airway infect	1.96	202.04	1.87
173	airway infect	1.98	374.93	1.90
215	asthma	10.37	456.16	0.18
**216**	**asthma**	21.75	1435.85	7.87
217	asthma	9.19	312.08	6.30
218	asthma	0.74	6685.65	21.67
220	asthma	8.10	1312.36	3.87
222	asthma	3.56	1916.28	6.94
224	asthma	0.53	861.78	0.00
236*	asthma	143.39	2204.84	253.09
242	asthma	4.20	2865.26	65.42
246	asthma	2.55	283.03	0.77
307	COPD	6.20	1168.00	11.61
313	COPD	20.84	1675.99	3.00
314	COPD	5.66	6827.54	32.36
315	COPD	4.67	103.67	1.24
**317**	**COPD**	35.59	18737.29	43.20
319	COPD	4.44	1125.41	2.19
**337**	**COPD**	31.82	19214.47	136.60
338	COPD	0.62	338.05	0.51
**339***	**COPD**	228.17	47374.78	386.09
340	COPD	2.27	1799.51	10.72
